# Case Report: FGFR2 inhibitor resistance via *PIK3CA* and *CDKN2A/B* in an intrahepatic cholangiocarcinoma patient with *FGFR2-SH3GLB1* fusion

**DOI:** 10.3389/fonc.2025.1527484

**Published:** 2025-04-07

**Authors:** Nadja Ballin, Alexander Ott, Olga Seibel-Kelemen, Irina Bonzheim, Dominik Nann, Janina Beha, Stephan Spahn, Stephan Singer, Stephan Ossowski, Cristiana Roggia, Christopher Schroeder, Michael Bitzer, Sorin Armeanu-Ebinger

**Affiliations:** ^1^ Institute of Medical Genetics and Applied Genomics, University Hospital Tübingen, Tübingen, Germany; ^2^ Department of Pathology and Neuropathology, University Hospital Tübingen, Tübingen, Germany; ^3^ Center for Personalized Medicine, University Hospital Tübingen, Tübingen, Germany; ^4^ Department of Internal Medicine I, University Hospital Tübingen, Tübingen, Germany

**Keywords:** FGFR inhibition, secondary resistance, PIK3CA, intrahepatic cholangiocarcinoma (iCCA), molecular tumor board, CDKN2A/B

## Abstract

*FGFR2* fusions occur in up to 14% of patients with intrahepatic cholangiocarcinoma (iCCA) and have been considered as therapeutic target for FGFR inhibitors (FGFRi). However, response to targeted treatment may be limited due to the emergence of various resistance mechanisms. We report a case of recurrent iCCA in a 43-year-old patient with a *FGFR2* fusion, who was treated with Lenvatinib. Next-generation sequencing (NGS) of tumor-normal DNA and tumor RNA under Lenvatinib treatment confirmed the *FGFR2* fusion, however no further molecular resistance mutation was observed. After failure of FGFRi treatment (Lenvatinib and Infigratinib) ten months later, repeated NGS analysis revealed a new gain-of-function mutation in *PIK3CA* and a homozygous deletion of *CDKN2A/B*, potentially representing an acquired resistance mechanism. The emerging acquired resistance to FGFR inhibitor treatment has implications for subsequent treatment strategies.

## Introduction

With the advent of precision medicine, the management of intrahepatic cholangiocellular carcinoma (iCCA) patients has been shaped in the last decade by molecular stratification of patients highlighting an enrichment of *FGFR* alterations in this tumor entity. Notably, oncogenic *FGFR2* alterations constitute in up to 14% of iCCA cases the main tumor driver (1–6). Consequently, a variety of FGFR inhibitors (FGFRi) have been developed and achieved approval by the FDA as second line therapy in patients with unresectable locally advanced or metastatic iCCA with *FGFR2* aberrations (7, 8). These are second generation FGFR inhibitors Pemigatinib and Infigratinib which are reversible competitive inhibitors of the ATP binding pocket of FGFR1-4 (9). Pemigatinib has also gained approval by the EMA. More recently, a third generation irreversible FGFR inhibitor, Futibatinib, has also been approved by the FDA and EMA for treatment of unresectable advanced or metastatic, chemotherapy refractory iCCA with *FGFR2* rearrangements (10, 11).

Although 23 - 42% of *FGFR2* fusion positive iCCA patients respond to FGFRi (7, 8, 11), secondary resistance and disease progression occur in most cases (9, 12). To date, different types of acquired resistance have been reported in iCCA patients after progression on FGFRi treatment. In up to 60% of cases resistance to FGFRi is mediated by secondary *FGFR* kinase domain mutations (13). Secondary resistance can also be mediated by acquired mutations with constitutive activation effect of FGFR2 itself and activation of alternate or downstream signaling pathways (MAPK or PIK3CA/MTOR). However, this type of acquired resistance has not been extensively studied in iCCA patients so far.

Here we present a patient with *FGFR2* fusion positive iCCA that developed resistance to FGFRi treatment. Sequential NGS analysis of tumor and normal DNA and tumor RNA reveal potential causes of acquired resistance, which have not been described in patients with iCCA up to date. This case highlights the need for broad tumor characterization to identify potential resistance mechanisms and additional treatment options.

## Case presentation

### Clinical course

**Figure 1:**
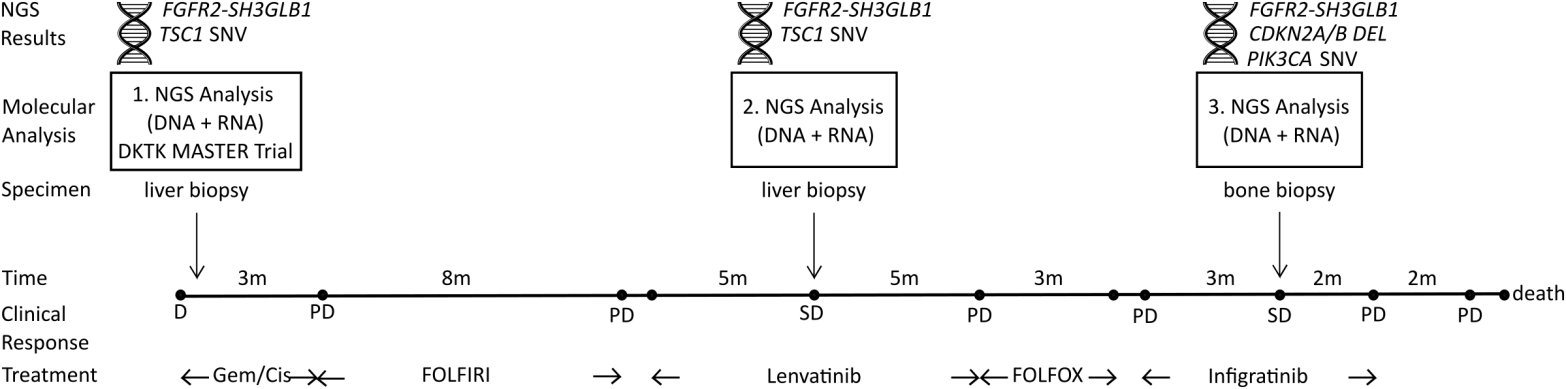
Clinical course and time points of three consecutive NGS analyses. First, DNA and RNA samples were obtained from a liver biopsy as part of DKTK MASTER Trial (NCT05852522) and NGS analysis identified a *FGFR2-SH3GLB1* fusion and a *TSC1* SNV as potential tumor drivers. The second NGS analysis was performed with DNA and RNA isolated from a liver biopsy during stable disease (SD) under Lenvatinib treatment. NGS results confirmed the FGFR2 fusion and *TSC1* SNV. Eleven months later, a third NGS analysis was performed on DNA and RNA from a bone biopsy during SD under Infigratinib treatment. The NGS results confirmed the FGFR2 fusion and identified a new *CDKN2A/B* deletion together with an activating *PIK3CA* mutation. SNV, small/single nucleotide variant; DEL, homozygous deletion; PD, progressive disease; D, initial diagnosis; Gem/Cis, Gemcitabine and Cisplatin treatment.

A 43-year-old patient presented at the department of internal medicine with hepatic, lymphogenic and pulmonary metastasized iCCA. He was first diagnosed with iCCA eleven months before and had already undergone treatment with gemcitabine/cisplatin (4 cycles, 3 months), followed by FOLFIRI (17 cycles, 8 months) (**Figure 1**). As part of his inclusion in the DKTK MASTER Trial (NCT05852522), the patient had received a first tumor DNA and RNA NGS analysis with identification of a *FGFR2-SH3GLB1* fusion, likely presenting the main tumor driver. Consequently, he started treatment with Lenvatinib after progression on FOLFIRI. Clinical response of this patient to Lenvatinib treatment and *in vitro* characterization of the *FGFR2-SH3GLB1* fusion have been reported in detail previously (14). After initial tumor regression, stable disease was observed after 5 months of treatment with Lenvatinib. At this point, a liver biopsy was taken. DNA and RNA were isolated and subjected to NGS analysis (panel sequencing of tumor/normal DNA and transcriptome sequencing of tumor RNA). Results were discussed in the local molecular tumor board with the recommendation of FGFR targeted therapy. Treatment with Lenvatinib was continued for 5 months until hepatic, lymphogenic and pulmonary progression was observed. Treatment was changed to FOLFOX for three months. Upon occurrence of new bone metastases, the patient was admitted to a phase II study with Infigratinib (BGJ398QED). After three months of treatment with Infigratinib stable disease was achieved with constant liver lesions, regression of pulmonary lesions and progression of bone metastases. Therefore, a bone biopsy was taken. NGS analysis of this specimen confirmed the presence of *FGFR2-SH3GLB1* fusion and revealed new drivers including an activating *PIK3CA* mutation and a homozygous deletion of *CDKN2A*. Infigratinib treatment was continued for two months until progression of bone metastases. The patient died three months after termination of Infigratinib treatment.

### Molecular analysis

At all-time points tested, the *FGFR2-SH3GLB1* fusion was detected in tumor DNA and RNA (**Supplementary Figure S1**). The fusion protein results from a translocation event t(1;10)(p22.3;q26.13) with genomic breakpoints detected in tumor DNA in intron 17 of *FGFR2* (ENST00000457416.7) and in intron 4 in *SH3GLB1* (ENST00000616170.4). Transcriptome analysis further confirmed that the translocation leads to an in-frame fusion of both genes. The putative fusion protein contains the N-terminal part of FGFR2 with an intact protein kinase domain fused to the C-terminal part of SH3GLB1 with a Bar domain and variant SH3 domain. Both the genomic breakpoint in *FGFR2* as well as the architecture of the FGFR2 fusion protein are typical of an oncogenic activation of FGFR2 via constitutive dimerization mediated by the BAR domain in the fusion partner SH3GLB1 (15). Therefore, *FGFR2-SH3GLB1* fusion was determined as the main driver in this iCCA and FGFRi treatment was recommended.

In addition to the *FGFR2-SH3GLB1* fusion, the first and second tumor-normal DNA analyses showed a likely oncogenic stop-gain variant in *TSC1* together with a heterozygous deletion of the unaffected allele in the liver biopsy (**Supplementary Figure S2**). However, the variant was only present in a fraction of tumor cells and was therefore considered a less-important therapeutic target. This variant was absent in the third tumor DNA analysis from a bone biopsy.

The third NGS analysis of tumor-normal DNA from a progressive bone biopsy taken under Infigratinib treatment revealed a different somatic single nucleotide variant (SNV) profile with an oncogenic, activating *PIK3CA* c.3140A>T, p.(His1047Leu) variant. This *PIK3CA* variant represents a known somatic hotspot variant typically found in breast cancer patients (cancerhotspot.org, COSMIC). Although *PIK3CA* His1047Leu variant is less frequently observed than His1047Arg, it has been shown to constitutively activate PI3K which is downstream of FGFR signaling (16). Additionally, the non-mutated allele was lost by a copy-neutral loss of heterozygosity (LOH) event in the tumor cells (**Supplementary Figure S2**). Therefore, this variant likely represented an acquired oncogenic driver in this tumor acting downstream of the oncogenic *FGFR2-SH3GLB1* fusion.

Comparison of the copy number variations (CNVs) between the two time points of the second and third NGS analysis performed at our institute showed an overall increase in number of CNVs (**Supplementary Figure S3**). The majority of these new CNVs were heterozygous deletions of complete or partial chromosome arms. On chromosome 9 a focal homozygous deletion of the *CDKN2A*/*CDKN2B* locus was detected in the bone biopsy, but not in the previous liver biopsies. This represented an additional acquired oncogenic event as the loss of *CDKN2A* leads to an activation of cell cycle (17).

## Discussion

Knowledge of the type of therapy resistance is extremely important as it determines subsequent treatment options.

Regarding FGFR inhibitors secondary resistance is most frequently mediated by an acquisition of gatekeeper mutations that sterically block the binding of competitive inhibitors to the ATP binding pocket in the kinase domain (18, 19). Other kinase domain mutations that confer resistance to selective FGFR inhibitors affect the FGFR2 autoinhibitory domain. The residues N549, E565 and K641 form a molecular brake and, upon disruption via point mutations, *FGFR2* becomes constitutively active (14, 20, 21). In this situation, switching to an irreversible FGFRi like Futibatinib or treatment with Lenvatinib as a multitargeted tyrosine kinase inhibitor may overcome therapy resistance (14, 22). In comparison to these direct mutations of FGFR, the activation of *PIK3CA* and loss of *CDKN2A/B* may induce resistance independent of FGFRi binding by activation of downstream signaling pathways.

**Figure 2:**
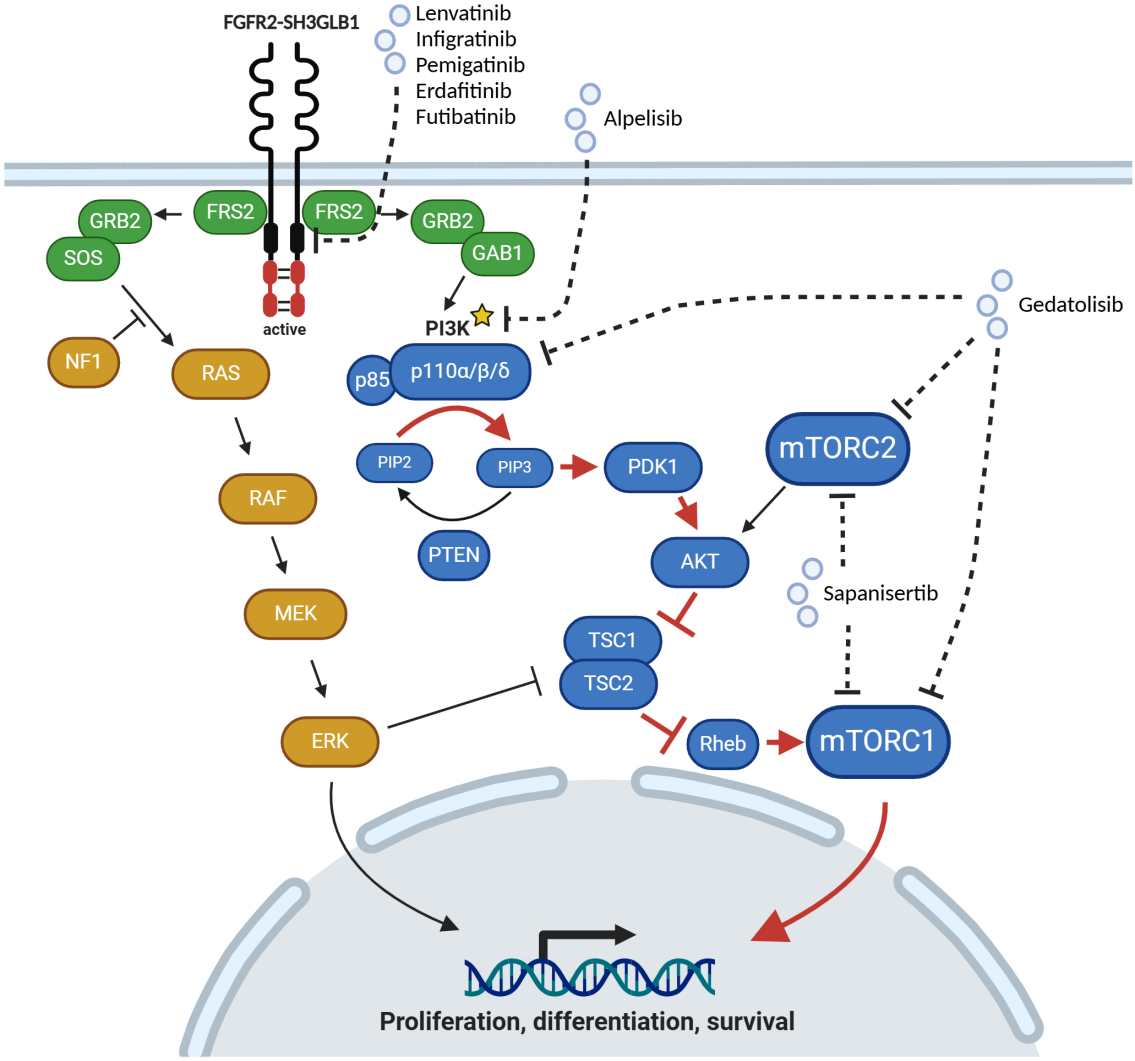
Activation of MAPK and PI3K/MTOR signaling pathways by oncogenic FGFR2 fusion protein. Upon inhibition of FGFR2 signaling by Lenvatinib and Infigratinib in this case of iCCA an activating *PIK3CA* (p110α) mutation (marked with an asterisk) was detected, which leads to an aberrant activation of downstream signaling pathway (red arrows). p110α can be targeted by Alpelisib. Alternatively, Gedatolisib, a pan-PI3K and dual mTOR inhibitor, or Sapanisertib, a dual mTOR inhibitor, could be used for targeted therapy. Created with BioRender.com.

Activation of a downstream or alternate signaling pathway may also confer resistance to targeted therapy/FGFR inhibition. Oncogenic FGFR2 fusions lead to a hyperactivation of FGFR2 signaling and consequently aberrant activation of RAS-RAF-ERK and PI3K-MTOR signaling, which promotes pro-tumorigenic processes such as proliferation, modulation of differentiation and survival (**Figure 2**). To escape the shutdown of these oncogenic signaling by FGFR inhibition tumors may gain activating alterations further downstream of the FGFR2 receptor. Yet, evidence for mutations activating downstream or alternate signaling pathways is still limited in iCCA patients.

So far, two independent studies of small cohorts of cholangiocarcinoma patients receiving FGFR targeted therapy reported the acquisition of MAPK or PI3K/MTOR pathway alterations. These alterations occurred in the majority of patients together with new FGFR mutations as secondary resistance (23, 24). Among these, three cases with *FGFR2* fusions gained changes in *PIK3CA* unlikely to constitutively activate the kinase under FGFRi treatment together with alterations in other pathways or FGFR2 itself (23) and one patient harbored a *PIK3CA* H1047R mutation at baseline examination as an additional oncogenic driver (24). Targeting MAPK pathway co-alterations in *KRAS* and *BRAF* to enhance efficacy of FGFR inhibition revealed synergism with MEK inhibitors Trametinib and Binimetinib, and AKT inhibitors *in vitro* (23). Similarly, a case report of a *FGFR2* altered iCCA patient showed secondary K/NRAS activation in addition to gatekeeper mutations in *FGFR2* upon progression on treatment with Pemigatinib (25). However, a sole activation of PI3K/MTOR pathway after resistance to FGFRi treatment has not been shown.

Here, we present a case of *FGFR2* fusion positive iCCA who acquired an oncogenic gain-of-function mutation in *PIK3CA* together with a homozygous deletion of *CDKN2A/B* upon treatment with Infigratinib and Lenvatinib. These alterations were detected by NGS analysis of a tumor biopsy taken during the treatment with Infigratinib. Consistent with the presence of a potential acquired resistance disease progression was observed two months later and targeting the resistance alterations as discussed in the molecular tumor board was not feasible.

The presence of new alterations in the bone metastasis could also be an expression of tumor heterogeneity. However, the bone metastasis progressed on FGFRi and was therefore accessible for analysis. In order to fully understand the causal relationship between the *PIK3CA* His1047Leu mutation and *CDKN2A/B* deletion in resistance to FGFRi treatment further functional studies in e.g. patient-derived organoids are needed.

Evidence for *PIK3CA* activation as a resistance mechanism to FGFR targeted therapy comes from FGFR driven urothelial cancer (26). Facchinetti et al. reported activation of MTOR signaling pathway in 58% (11/19) of FGFRi (Futibatinib or Erdafitinib) treated urothelial cancer patients with *FGFR* alterations. Two of these patients acquired an activating *PIK3CA* mutation at position E545K and E726K after progression on Erdafitinib treatment. Furthermore, they could show sensitivity of a patient derived xenograft with FGFR alteration and *PIK3CA* E545K to the combination treatment with Erdafitinib and Pictilisib (PI3K inhibitor). In addition, Arai et al. reported urothelial cell lines treated with Erdafitinib to gain resistance by *PIK3CA* mutation (27).

Moreover, activation of PI3K is an established mechanism of primary resistance to upstream HER2-targeted therapy in HER2+ breast cancer patients (28). In the SOLAR-1 study, HR+ HER2- advanced or metastatic breast cancer patients benefited from addition of PI3K inhibitor Alpelisib to Fulvestrant treatment if they harbored a *PIK3CA* mutation. Consequently, Alpelisib in combination with Fulvestrant gained FDA and EMA approval for this group of *PIK3CA*-mutated breast cancer patients. In FGFR2 rearranged iCCA, three of nine patients with *PIK3CA* mutation presented with concomitant inactivation of *CDKN2A* and had stable disease (one patient) as best response to Pemigatinib treatment (29).

Along with the gain of function variant in *PIK3CA* a homozygous deletion of *CDKN2A* and *CDKN2B* was detected by NGS analysis as an additional potentially acquired oncogenic event in our case of FGFR driven iCCA. FGFR signaling and *CDKN2A/B* both drive cell cycle progression via inactivation of p27 (30, 31), which supports our observation of emerging *CDKN2A/B* loss upon FGFRi treatment as potential resistance mechanism. Furthermore, co-occurrence of inactivation of *CDKN2A/B* with FGFR2 rearrangement significantly reduced median PFS in iCCA patients treated with Pemigatinib (29) and emphasizes the need for combination therapy. Interestingly, deletion of *CDKN2A* has been reported as concurrent resistance mechanism in two NSCLC patients treated with Abivertinib, a third generation tyrosine kinase/EGFR inhibitor (32). In these two cases *CDKN2A* deletion was detected together with acquired *EGFR* amplification. PI3K/mTOR pathway activation and cell cycle dysregulation jointly drive resistance (33). In breast cancer cells a combinatorial drug screening found that CDK4/6 inhibition exhibits the most significant synergism with PI3K inhibitors (34).

Dual oncogenic activation of signaling pathways argues for application of combination therapy. In this case, with three oncogenic drivers, in theory several combinations might be interesting.

Focusing on the *FGFR* fusion and activation of mTOR signaling by the *PIK3CA* alteration, combination of Infigratinib with an PIK3CA inhibitor (e.g. Alpelisib) would have been an option. This combination showed a manageable safety profile in a first in-human combination trial (35), however frequent dose interruptions or reductions were necessary and potentially limit their long-term application. A first assessment of synergistic effects in patients with solid tumors harboring *PIK3CA* and/or *FGFR1-3* alterations failed to show any link to this molecular subtype. Yet, in respect to *FGFR2* alterations this study was restricted to SNVs/Indels, which may already confer resistance to Infigratinib.

Interestingly, *in vitro* combination therapy of a FGFR2 fusion positive cell line with FGFR2 E565A molecular brake mutation with Infigratinib and dual mTOR inhibitor Sapanisertib (CB-228, formerly TAK-228/MLN0128) was able to reverse resistance to Infigratinib (21). Moreover, Facchinetti et al. reported one iCCA patient with a FGFR2 fusion together with *PIK3CA* H1047R activating mutation with progression on Pemigatinib treatment, who, however, benefited from second line treatment with Everolimus. Administration of Everolimus also showed anti-tumor activity in two phase II studies including iCCA patients (36, 37) and in a case report of *PIK3CA* E545G mutated iCCA patient (38).

Hence, it remains possible that mTOR inhibition or more specific PIK3CA inhibition would have been able to resensitize the tumor of the presented patient to Infigratinib treatment.

Considering the concomitant occurrence of an inactivation of *CDKN2A*, combination treatment with a CDK4/6 inhibitor (e.g. Palbociclib or Ribociclib) may also be relevant. From a mechanistic perspective, triple combination of a CDK4/6 inhibitor with an FGFR inhibitor and a PI3K/MTOR inhibitor might be ideal. However, clinical applicability of such a combination still has to be evaluated. So far, a phase Ib trial of Fulvestrant with Palbociclib and Erdafitinib in FGFR-amplified/ER+/HER2-negative metastatic breast cancer has been conducted and has reported on-target toxicities for Erdafitinib leading to treatment discontinuation (39). Combined inhibition of mTOR and CDK4/6 has been positively evaluated in patients with advanced breast cancer showing acceptable safety profiles. In this context, in a phase I/II trial of patients with ER+/HER2-negative metastatic breast cancer who had progressed on CDK4/6 targeted therapy the triplet combination of Ribociclib, Everolimus and Exemestane showed an acceptable safety profile (40). In addition, combination of PI3K/mTOR inhibitor Gedatolisib plus Palbociclib and endocrine therapy in women with HR+/HER2-negative advanced breast cancer was well tolerated with an acceptable safety profile (41). In the context of iCCA, pre-clinical studies have reported synergistic effects of combined CDK4/6 and mTOR inhibition (42).

To sum up, the presented case highlights the importance of sequential broad molecular tumor characterization in order to capture the full spectrum of potential resistance mechanisms to targeted therapy. Here, oncogenic changes in *PIK3CA* and *CDKN2A/B* were identified after progression on Infigratinib/Lenvatinib treatment. These are in principle targetable alterations that could be addressed by different combination therapies. Manageable safety profiles of drug combinations with maintenance of good quality of life in such complex molecularly altered tumors still require further assessment.

## Methods

### Patient

The patient provided written informed consent for blood collection, tissue biopsies, NGS analysis, treatment and publication of this report.

NGS analysis was performed in a diagnostic setting and already published in detail under (43–46).

### DNA and RNA isolation

Genomic DNA was isolated from normal tissue (peripheral blood) using Qiagen FlexiGene (Qiagen, Hilden, Germany). Tumor DNA and RNA were extracted from macrodissected 5μm paraffin sections using the Maxwell RSC DNA FFPE Kit and Maxwell RSC RNA FFPE Kit and the Maxwell RSC Instrument (Promega, Madison, WI, USA) according to the manufacturer’s instructions. DNA concentrations were determined using the Qubit™ DNA BR kit (Thermo Fisher Scientific, Waltham, MA, USA) and DNA integrity was analyzed using the Agilent Genomic DNA Screen Tape System (Agilent, Santa Clara, CA, USA).

### Next generation sequencing (NGS)

For each DNA sample 200 ng genomic DNA was sheared to 150-200 bp length using a Covaris E220 Ultrasonicator (Covaris, Woburn, MA, USA). Fragmented DNA was end repaired, A-tailed, adapter ligated and amplified with Agilent’s SureSelect XT Low Input Target Enrichment System for Illumina Paired-End Multiplexed Sequencing Library kit following the manufacturer’s instructions (Agilent Technologies, Santa Clara, CA, USA). Target enrichment was performed using a custom-designed hybrid capture panel covering 708 cancer-related genes, 7 promoter regions and selected fusions as well as microsatellite loci (Agilent Technologies, Santa Clara, CA, USA). The libraries were sequenced on a NovaSeq6000 sequencing platform (Illumina, San Diego, CA, USA) in paired-end mode as specified by the manufacturer.

Total tumor RNA was prepared for sequencing using New England Biolabs NEBNext Ultra II Directional RNA rRNA Depletion FFPE Kit according to the manufacturer’s instructions. Libraries were sequenced on a NovaSeq6000 sequencing platform (Illumina, San Diego, CA, USA) in paired-end mode.

### Bioinformatic data analysis and variant interpretation

An in-house bioinformatics pipeline megSAP (https://github.com/imgag/megSAP) was used to analyze NGS data. Both tumor and normal samples were mapped with bwa mem2 (47), ABRA2 (48) for INDEL realignment and SeqPurge (49) for adapter trimming followed by variant calling with Freebayes (50) for the germline sample and Strelka2 (51) for the differential analysis, respectively. Variant annotation was performed using variant effect predictor (VEP) and various damage scores. Germline and somatic copy number variation (CNV) calling was performed with ClinCNV (52).

Our decision support application GSvar (https://github.com/imgag/ngs-bits) was used for variant interpretation and generation of final reports. Somatic variants were classified according to Variant Interpretation for Cancer Consortium (VICC) (53).

### Transcriptome analysis

RNA data were demultiplexed using Illumina bcl2fastQ and then analyzed by our in-house bioinformatics pipeline megSAP. Normalized gene expression values FPKM (fragments per kilobase per million) and TPM (transcripts per kilobase per million) were calculated with Subreads Software (54). Fusions were identified with STAR-Fusion (55) and ARRIBA (56).

## Data Availability

Requests to access these datasets should be directed to NB, nadja.ballin@med.uni-tuebingen.de.
