# Supplementary material for: Case Report: FGFR2 inhibitor resistance via PIK3CA and CDKN2A/B in an intrahepatic cholangiocarcinoma patient with FGFR2-SH3GLB1 fusion
Source: Front Oncol. 2025 Apr 7;15:1527484. doi: 10.3389/fonc.2025.1527484 (PMC12009697; doi:10.3389/fonc.2025.1527484)
Supplement: Supplementary file 1 [file Presentation1.pptx]

## Slide 1
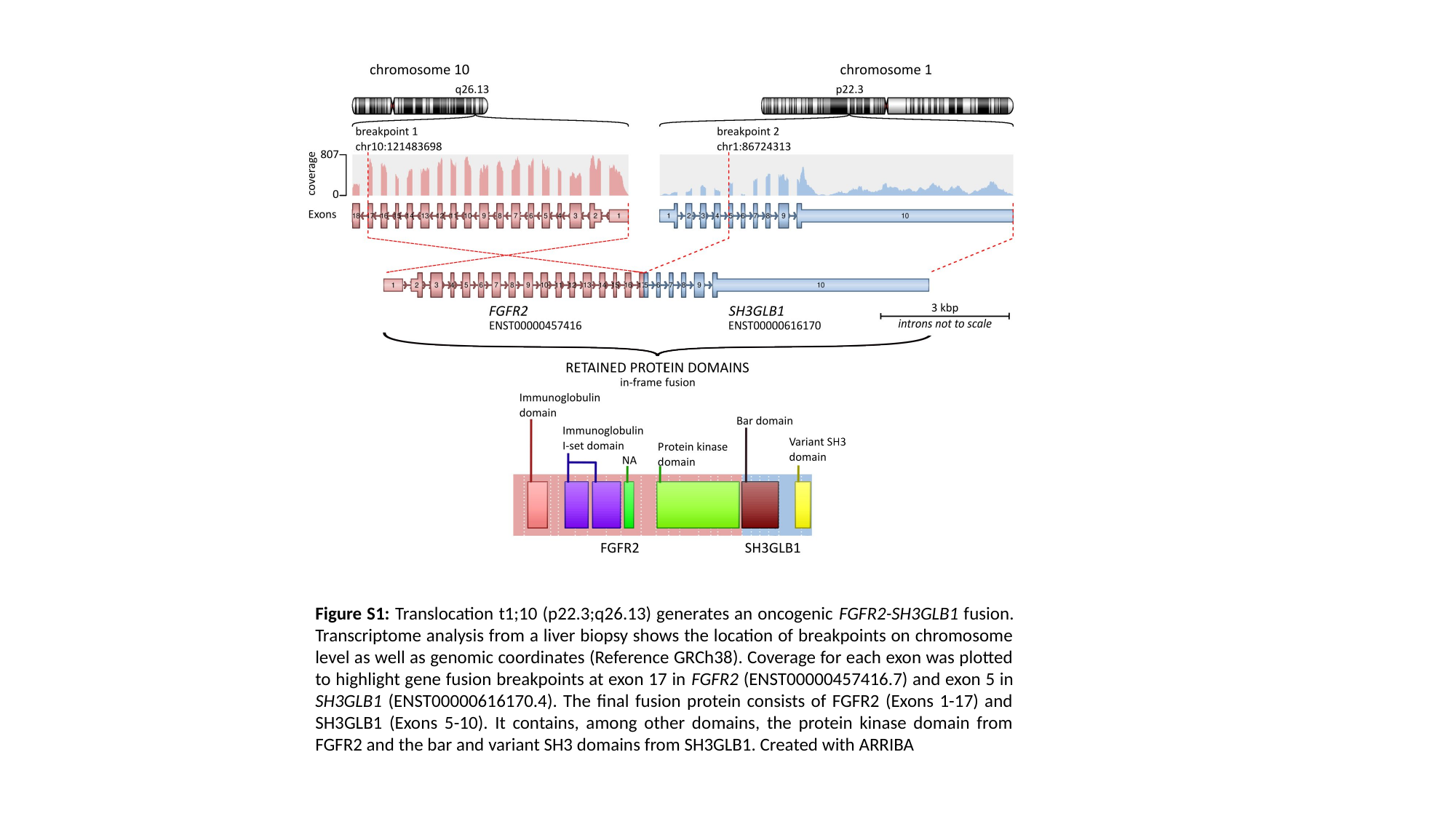

Figure S1: Translocation t1;10 (p22.3;q26.13) generates an oncogenic FGFR2-SH3GLB1 fusion. Transcriptome analysis from a liver biopsy shows the location of breakpoints on chromosome level as well as genomic coordinates (Reference GRCh38). Coverage for each exon was plotted to highlight gene fusion breakpoints at exon 17 in FGFR2 (ENST00000457416.7) and exon 5 in SH3GLB1 (ENST00000616170.4). The final fusion protein consists of FGFR2 (Exons 1-17) and SH3GLB1 (Exons 5-10). It contains, among other domains, the protein kinase domain from FGFR2 and the bar and variant SH3 domains from SH3GLB1. Created with ARRIBA

## Slide 2
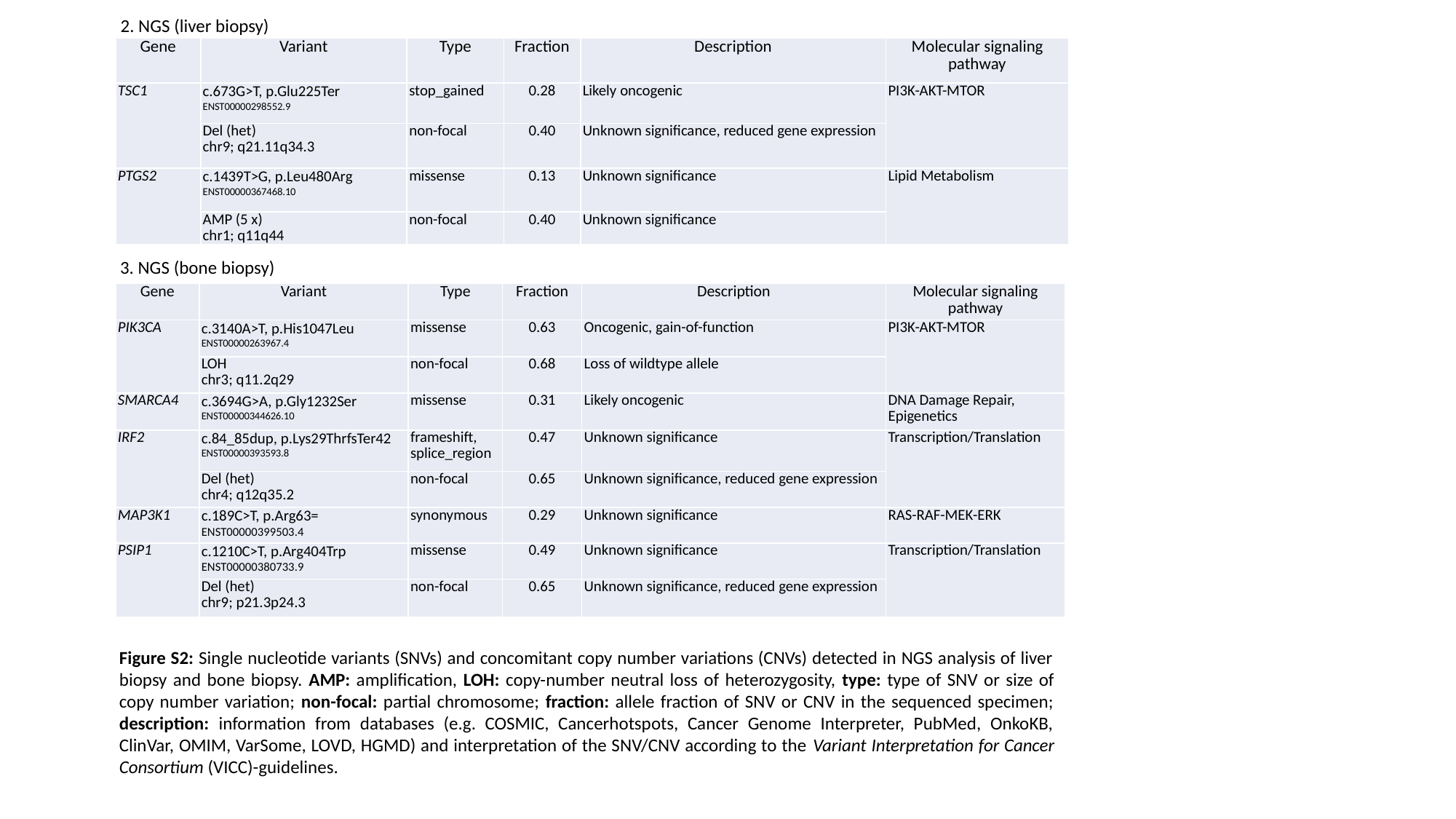

2. NGS (liver biopsy)
| Gene | Variant | Type | Fraction | Description | Molecular signaling pathway |
| --- | --- | --- | --- | --- | --- |
| TSC1 | c.673G>T, p.Glu225Ter ENST00000298552.9 | stop\_gained | 0.28 | Likely oncogenic | PI3K-AKT-MTOR |
| | Del (het) chr9; q21.11q34.3 | non-focal | 0.40 | Unknown significance, reduced gene expression | |
| PTGS2 | c.1439T>G, p.Leu480Arg ENST00000367468.10 | missense | 0.13 | Unknown significance | Lipid Metabolism |
| | AMP (5 x) chr1; q11q44 | non-focal | 0.40 | Unknown significance | |
3. NGS (bone biopsy)
| Gene | Variant | Type | Fraction | Description | Molecular signaling pathway |
| --- | --- | --- | --- | --- | --- |
| PIK3CA | c.3140A>T, p.His1047Leu ENST00000263967.4 | missense | 0.63 | Oncogenic, gain-of-function | PI3K-AKT-MTOR |
| | LOH chr3; q11.2q29 | non-focal | 0.68 | Loss of wildtype allele | |
| SMARCA4 | c.3694G>A, p.Gly1232Ser ENST00000344626.10 | missense | 0.31 | Likely oncogenic | DNA Damage Repair, Epigenetics |
| IRF2 | c.84\_85dup, p.Lys29ThrfsTer42 ENST00000393593.8 | frameshift, splice\_region | 0.47 | Unknown significance | Transcription/Translation |
| | Del (het) chr4; q12q35.2 | non-focal | 0.65 | Unknown significance, reduced gene expression | |
| MAP3K1 | c.189C>T, p.Arg63= ENST00000399503.4 | synonymous | 0.29 | Unknown significance | RAS-RAF-MEK-ERK |
| PSIP1 | c.1210C>T, p.Arg404Trp ENST00000380733.9 | missense | 0.49 | Unknown significance | Transcription/Translation |
| | Del (het) chr9; p21.3p24.3 | non-focal | 0.65 | Unknown significance, reduced gene expression | |
Figure S2: Single nucleotide variants (SNVs) and concomitant copy number variations (CNVs) detected in NGS analysis of liver biopsy and bone biopsy. AMP: amplification, LOH: copy-number neutral loss of heterozygosity, type: type of SNV or size of copy number variation; non-focal: partial chromosome; fraction: allele fraction of SNV or CNV in the sequenced specimen; description: information from databases (e.g. COSMIC, Cancerhotspots, Cancer Genome Interpreter, PubMed, OnkoKB, ClinVar, OMIM, VarSome, LOVD, HGMD) and interpretation of the SNV/CNV according to the Variant Interpretation for Cancer Consortium (VICC)-guidelines.

## Slide 3
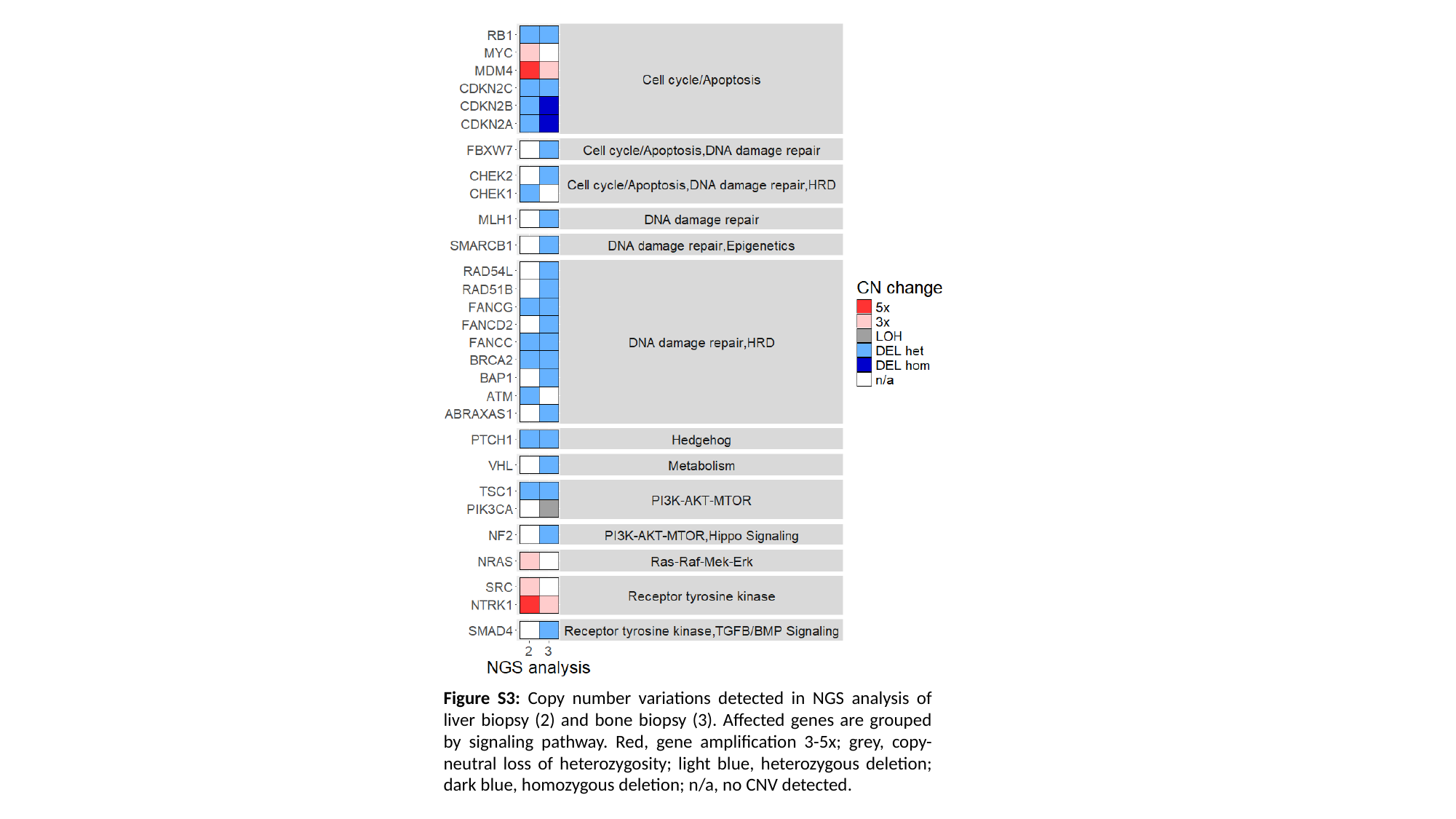

Figure S3: Copy number variations detected in NGS analysis of liver biopsy (2) and bone biopsy (3). Affected genes are grouped by signaling pathway. Red, gene amplification 3-5x; grey, copy-neutral loss of heterozygosity; light blue, heterozygous deletion; dark blue, homozygous deletion; n/a, no CNV detected.
